# Quantitative wound ballistic analysis of gelatin head phantoms by computed tomography using the total crack length method

**DOI:** 10.1007/s12024-025-00995-9

**Published:** 2025-03-20

**Authors:** Vasiliki Chatzaraki, Dominic Gascho, Michael J. Thali, Beat P. Kneubuehl, Carlo Tappero, Stephan A. Bolliger

**Affiliations:** 1 Institute of Forensic Medicine, University of Zurich, Zurich, CH-8057 Switzerland; 2 bpk consultancy gmbh, CH-3603 Thun, Switzerland

**Keywords:** Computed tomography, CT, Total crack length, Wound ballistics, Ordnance gelatin, Forensic imaging

## Abstract

Gelatin-based phantoms are appropriate simulants of human soft tissue and can be used for ballistic experiments. Computed tomography (CT) is useful for quantitative wound ballistics analysis and has been applied to gelatin blocks. This study aimed to create total crack length (TCL) profiles along the penetration depth in head phantoms after ballistic experiments with different types of ammunition at varying distances using CT data. Sixteen commercially available gelatin-based head phantoms were subjected to ballistic testing with six different types of ammunition. Nine were contact shots, while seven were fired from a 15-meter distance. Following the experiments, CT scans of the phantoms were performed. The TCL, a secondary effect of the temporary cavity, was measured based on the CT data. All phantoms exhibited perforation shots. The CT data enabled measurements along the penetration depth for each phantom. Metal artifacts caused by small projectile fragments along the path did not interfere with the measurements. The resulting TCL curves for the different ammunition types demonstrated distinct differences. TCL measurements in gelatin-based head phantoms are feasible and provide values proportional to the transferred energy at a given penetration depth for different ammunition types and shooting distances.

**Supplementary Information:**

The online version contains supplementary material available at 10.1007/s12024-025-00995-9.

## Introduction

Ordnance gelatin and glycerin soap are widely recognized as appropriate simulants of human tissue and have proven valuable in wound ballistics research. Ballistic glycerin soap undergoes plastic deformation, effectively “freezing” the maximum expansion of the temporary cavity. By measuring the volume of this cavity, the transferred energy can be calculated using a proportionality constant [1]. In contrast, gelatin exhibits elastic deformation, ultimately revealing the permanent wound channel observed in real gunshot wounds. Additionally, when the temporary cavity expands significantly, radial cracks may form [1]. These cracks provide further insights into the energy transfer process [2–4]. Traditionally, gelatin is sliced to measure these cracks; however, this method risks introducing undesirable artifacts such as crack elongation or inconsistent slice thickness.

Computed tomography (CT) offers a non-invasive alternative for measuring volumes, penetration depths, cross-sectional areas, and crack lengths, presenting advantages over conventional slicing techniques [3, 5–15]. For instance, Korać et al. [5] demonstrated that CT data could be used to precisely measure the cross-sectional areas of projectile paths and the volume of permanent wound channels in silicone blocks. This suggests that with modern thin-slice CT scanners, the effects, discrepancies, and cavity behaviors of various ammunition types can be analyzed with submillimeter accuracy at consistent penetration depths. To estimate wounding potential, it is essential to determine the volume of the temporary cavity at regular intervals along the penetration path, typically every centimeter, as this correlates with the energy transferred at each specific depth [1]. One method for inferring the extent of the temporary cavity in gelatin is total crack length (TCL) measurement [1]. Such TCL measurements along the projectile path in ordnance gelatin reflect the energy transfer profile specific to each type of ammunition used [2, 3]. These measurements can be performed visually, as shown in photographs of traditionally sliced gelatin by Ragsdale et al. [2], or radiologically, using CT images of virtually sliced gelatin, as demonstrated by Bolliger et al. [3]. When ballistic experiments involve head phantoms, the presence of a bone simulant around the gelatin can introduce additional structural damage, making CT-based analysis particularly advantageous. Thali et al. [16] previously demonstrated the feasibility of examining head phantoms using CT. However, to our knowledge, no studies have yet used the CT-based TCL method to assess the extent of temporary cavity formation in head phantoms for various common ammunition types.

The purpose of this study was to apply CT scanning to head phantoms following ballistic experiments with different ammunition types at two different distances and to create TCL profiles along the penetration depth based on the CT data.

## Materials and methods

The conduct of this experimental study was authorized by the Chair of Forensic Medicine of the University.

Sixteen commercially available gelatin-based head phantoms and six different types of ammunition were used in this study:

- 22 long rifle (LR) lead round nose.
- 5.56 mm North Atlantic Treaty Organization (NATO).
- 7.5 mm *Gewehrpatrone* (GP) 11 (Swiss).
- 9 mm Luger full metal jacketed (FMJ) round head.
- 9 mm Luger Action 4.
- 44 Remington Magnum semi-jacketed hollow point (SJHP).

**Fig. 1:**
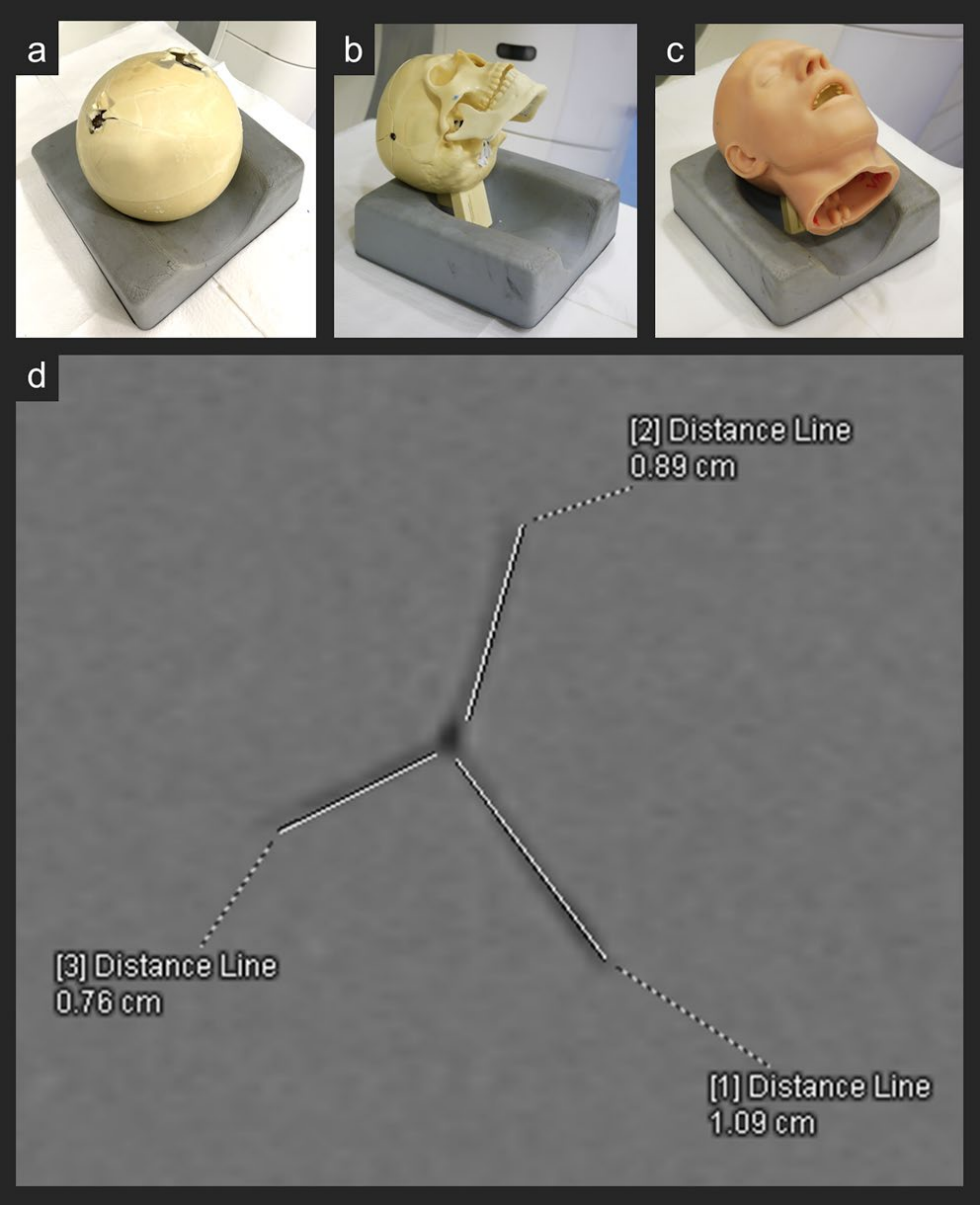
Bone simulants in spherical shape (**a**) or anatomical skull form without (**b**) and with additional skin simulant (**c**) were filled with gelatin. The transverse CT slice (zoomed image) shows the measurement of the crack lengths along the projectile path of a 22 long rifle (LR) through the gelatin inside such a phantom (**d**). The figure shows the length measurements (white lines) of the visible cracks (displayed in dark) using the distance tool of the CT viewing software. In this case, three cracks were measured, which can be recognized by the three white lines. In total, the crack lengths at the respective penetration depth result in a value of 2.74 cm

Each phantom was filled with 10% ordnance gelatin at 4 °C and encased in a bone simulant. Three different configurations were used: spherical (Fig. 1.a), anatomical skull form without an additional skin simulant (Fig. 1.b), and anatomical skull form with an additional skin simulant (Fig. 1.c). A total of 16 shots were performed: nine contact shots and seven from a 15-meter distance. An overview is provided in Supplementary Material 1.

All head phantoms were scanned using CT within 12 h of the shooting experiments. The CT examinations were performed using a 128-slice CT scanner (SOMATOM^®^ Definition Flash, Siemens Healthineers, Erlangen, Germany) with the following parameters: 1000 mAs (effective), 120 kV, pitch 0.35, and a rotation time of 1 s. Image reconstruction was carried out with a standard hard kernel (Hr60), using a slice thickness of 0.6 mm and a field of view of 300 × 300 mm^2^ (at a matrix resolution of 512 × 512), generating nearly isotropic voxels of 0.6 mm^3^.

The TCL along the projectile paths was radiologically measured using *Syngo.via* imaging software for multimodality reading (*Syngo.via*, Siemens Healthineers, Erlangen, Germany). Multi-planar reconstruction was used to align the CT dataset along the projectile path, enabling TCL measurements in cross-sections (Fig. 1.d). The TCL per slice was measured every 10 mm, corresponding to the distance between images along the penetration depth. A detailed description of the measurement procedure is available in Supplementary Material 2. Finally, the TCL per slice was plotted as a function of penetration depth. A moving mean curve was plotted to visualize the entire TCL distribution along the projectile path. This curve was based on the mean values of two consecutive slices, employing a statistical smoothing method for the dataset. The quantitative and visual analysis of the measured values was conducted using *Microsoft Excel* (© 2016 Microsoft Corporation, USA). In addition, the CT images were examined for metallic fragments to determine partial perforation, meaning whether larger fragments were visible. Proper reconstruction windowing was applied to enhance visualization and minimize radiation streak artifacts. All measurements and image analyses were performed by a forensic pathologist with seven years of experience in forensic radiology at the time of image review.

To assess data normality, the Shapiro-Wilk test was used with a significance level of *p* ≤ 0.05. The Kruskal-Wallis rank sum test was conducted to determine whether the projectiles differed significantly in their TCL values (significance level: *p* ≤ 0.05). Dunn’s test with Bonferroni correction was applied as a post hoc test to identify significant differences between specific pairs of ammunition types. All statistical analyses were performed using the *RStudio* programming environment for statistical computing and graphics (*RStudio*, Inc., Boston, MA, USA).

## Results

All experiments resulted in perforation shots, meaning no lodged projectiles were detected on the CT images and only small projectile fragments were visible along the path. The metal artifacts caused by these fragments did not impede crack length measurements.

**Fig. 2:**
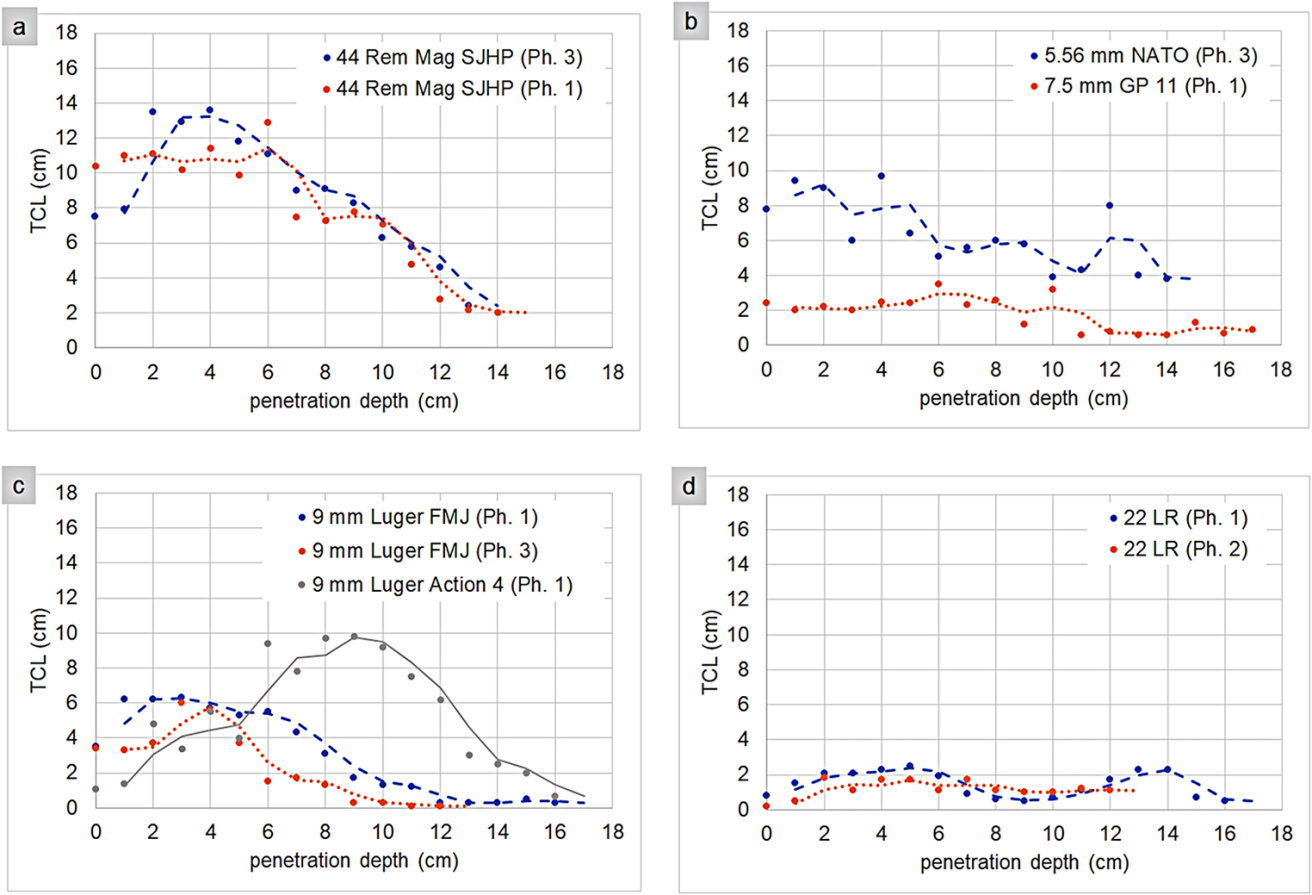
Contact shots: TCL (y-axis) as a function of the projectile penetration depth (x-axis) to visualize the overall TCL curve (moving mean) for the 44 Remington Magnum projectiles (**a**), the 5.56 mm NATO and the 7.5 mm GP 11 projectile (**b**), the 9 mm Luger projectiles (**c**), and the 22 LR projectiles (**d**). The individual data points in this line chart represent the sum of the crack lengths on the respective CT slice, which corresponds to a certain penetration depth (CT slice 1: 0 cm penetration depth, CT slice 2: 1 cm penetration depth, CT slice 3: 2 cm penetration depth, and so on). Rem = Remington, Mag = Magnum, SJHP = semi-jacketed hollow point, NATO = North Atlantic Treaty Organization, GP = *Gewehrpatrone*, FMJ = full metal jacket, LR = long rifle. Ph = phantom, phantom 1 = head-sphere phantom, phantom 2 = head-skull phantom, phantom 3 = head-skull phantom with synthetic skin

**Fig. 3:**
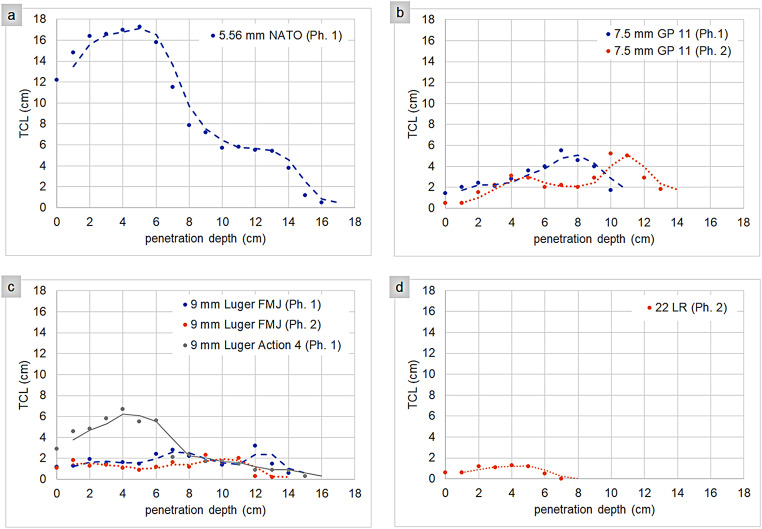
Long-range shots: TCL (y-axis) as a function of the projectile penetration depth (x-axis) to visualize the overall TCL curve (moving mean) for the 5.56 mm NATO projectile (**a**), the 7.5 mm GP 11 projectiles (**b**), the 9 mm Luger projectiles (**c**), and the 22 LR projectile (**d**). The individual data points in this line chart represent the sum of the crack lengths on the respective CT slice, which corresponds to a certain penetration depth (CT slice 1: 0 cm penetration depth, CT slice 2: 1 cm penetration depth, CT slice 3: 2 cm penetration depth, and so on). NATO = North Atlantic Treaty Organization, GP = Gewehrpatrone, FMJ = full metal jacket, LR = long rifle, Ph = phantom, phantom 1 = head-sphere phantom,phantom 2 = head-skull phantom (without synthetic skin simulant).

The CT-based TCL measurements for the different ammunition types along the projectile path are presented in Table 1 (contact shots) and Table 2 (distance shots). Based on these individual CT measurements, TCL curves along the projectile path are illustrated in Fig. 2 (contact shots) and Fig. 3 (distance shots).

**Table 1:** Contact shots: TCL values of the individual projectiles measured on the individual CT images, with a spacing of 1 cm between images, corresponding to a penetration depth from 0 to a maximum of 17 cm in 1 cm increments

Shot No.	Projectile	Penetration length (cm)																	
		0	1	2	3	4	5	6	7	8	9	10	11	12	13	14	15	16	17
1	44 Remington Magnum SJHP (3)	7.5	7.9	13.5	12.9	13.6	11.8	11.1	9	9.1	8.3	6.3	5.8	4.6	2.4	-	-	-	-
2	44 Remington Magnum SJHP (1)	10.4	11	11.1	10.2	11.4	9.9	12.9	7.5	7.3	7.8	7.1	4.8	2.8	2.2	2	-	-	-
3	5.56 mm NATO (3)	7.8	9.4	9	6	9.7	6.4	5.1	5.6	6	5.8	3.9	4.3	8	4	3.8	-	-	-
4	9 mm Luger FMJ (1)	3.5	6.2	6.2	6.3	5.7	5.3	5.5	4.3	3.1	1.7	1.3	1.2	0.3	0.3	0.3	0.5	0.3	-
5	9 mm Luger FMJ (3)	3.4	3.3	3.7	6	5.6	3.7	1.5	1.7	1.3	0.3	0.3	0.1	0.1	-	-	-	-	-
6	9 mm Luger Action 4 (1)	1.1	1.4	4.8	3.4	5.5	4	9.4	7.8	9.7	9.8	9.2	7.5	6.2	3	2.5	2	0.7	-
7	7.5 mm GP11 (1)	2.4	2	2.2	2	2.5	2.4	3.5	2.3	2.6	1.2	3.2	0.6	0.8	0.6	0.6	1.3	0.7	0.9
8	22 LR LNR (1)	0.8	1.5	2.1	2.1	2.3	2.5	1.9	0.9	0.6	0.5	0.7	1.1	1.7	2.3	2.3	0.7	0.5	-
9	22 LR LNR (2)	0.2	0.5	1.8	1.1	1.7	1.7	1.1	1.7	1.1	1	1	1.2	1.1	-	-	-	-	-

TCL = total crack length, CT = computed tomography, SJHP = semi-jacketed hollow point, NATO = North Atlantic Treaty Organization, FMJ = full metal jacket, GP = Gewehrpatrone, LR = long rifle, LNR = lead round nose. No value (-) means that the projectile has exited the phantom. Note: The number in brackets next to the projectile designation indicates the phantom: 1 = spherical head phantom, 2 = skull phantom, 3 = skull phantom with skin simulant

**Table 2:** Long-range shots: TCL values of the individual projectiles measured on the individual CT images, with a spacing of 1 cm between images, corresponding to a penetration depth from 0 to a maximum of 16 cm in 1 cm increments

Shot No.	Projectile	Penetration length (cm)																
		0	1	2	3	4	5	6	7	8	9	10	11	12	13	14	15	16
10	5.56 mm NATO (1)	12.2	14.8	16.4	16.6	17	17.3	15.8	11.5	7.9	7.2	5.7	5.8	5.5	5.4	3.8	1.2	0.5
11	9 mm Luger FMJ (1)	1.2	1.3	1.9	1.5	1.6	1.5	2.4	2.8	2.2	1.7	1.4	1.5	3.2	1.5	0.6	-	-
12	9 mm Luger FMJ (2)	1.1	1.8	1.3	1.4	1.1	0.9	1.2	1.6	1.2	2.3	1.6	2	0.3	0.2	-	-	-
13	9 mm Luger Action 4 (1)	2.9	4.6	4.8	5.8	6.7	5.5	5.6	2.1	2.4	1.7	1.7	1.5	0.9	0.9	0.9	0.3	-
14	7.5 mm GP11 (1)	1.4	2	2.4	2.1	2.8	3.6	4	5.5	4.6	4	1.7	-	-	-	-	-	-
15	7.5 mm GP11 (2)	0.5	0.5	1.5	2.2	3.1	2.9	2	2.2	2	2.9	5.2	5	2.9	1.8	-	-	-
16	22 LR LRN (2)	0.6	0.6	1.2	1.1	1.3	1.2	0.5	0	-	-	-	-	-	-	-	-	-

TCL = total crack length, CT = computed tomography, NATO = North Atlantic Treaty Organization, FMJ = full metal jacket, GP = Gewehrpatrone, LR = long rifle, LNR = lead round nose. No value (-) means that the projectile has exited the phantom. Note: The number in brackets next to the projectile designation indicates the phantom: 1 = spherical head phantom, 2 = skull phantom (without skin simulant)

In contact shots, the 44 Remington Magnum SJHP exhibited the highest TCL values within the first few centimeters after entry, followed by a steep decline over the remaining penetration depth toward the exit. The 5.56 mm NATO, which showed the second-highest TCL values in the initial few centimeters, maintained a more gradual decrease along the entire projectile path. Notably, the 7.5 mm GP 11 displayed relatively low TCL values, resulting in a flatter TCL curve comparable to that of the 22 LR projectiles. Among the 9 mm Luger projectiles, the FMJ initially exhibited slightly higher TCL values than the Action 4. However, after a few centimeters, the FMJ values declined, whereas the Action 4 values steadily increased, peaking at around 8 to 10 cm, twice as high as the FMJ maxima. The additional skin simulant had minimal influence on the TCL values between the two FMJ projectiles.

In long-range shots, the 5.56 mm NATO displayed by far the highest TCL values in the first few centimeters compared to the other ammunition types. However, no data were available for the 44 Remington Magnum SJHP in this test. After the initial peak, TCL values for the 5.56 mm NATO rapidly declined. Unlike in contact shots, the 7.5 mm GP 11 showed a clear increase in TCL values after a few centimeters, followed by a gradual decline. As in contact shots, the 22 LR displayed low TCL values. Its projectile path was also relatively short, as the phantom was not struck centrally. In contrast to contact shots, the 9 mm Luger FMJ did not exhibit high TCL values upon entering the gelatin. However, the 9 mm Luger Action 4 did show high TCL values immediately after entry, although these maximum values were significantly lower than those recorded in contact shots.

The Shapiro-Wilk test indicated that data for the 9 mm Luger FMJ (*p* = 0.0095) and 22 LR (*p* = 0.0243) were not normally distributed for contact shots on the spherical head model. Consequently, the Kruskal-Wallis test was used for further analysis, revealing a significant difference (χ² = 123.95, df = 15, *p* < 2.2e-16). The Dunn’s test with Bonferroni correction identified significant differences in 33 out of 120 possible projectile-phantom-distance pairs regarding the rank sum of TCL values. However, no significant differences were observed between contact and distance shots or between different phantoms for the same projectile type. Detailed results from Dunn’s test with Bonferroni correction are provided in Supplementary Material 3.

## Discussion

**Fig. 4:**
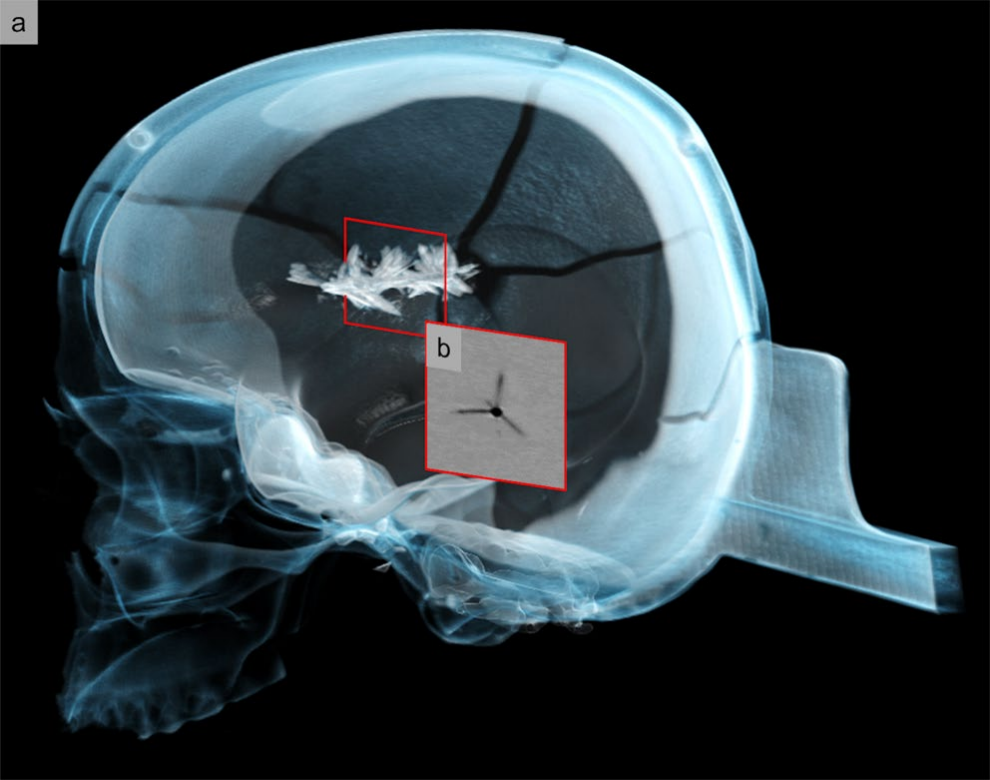
Three-dimensional visualization (volume rendering) and exemplary cross-sectional image of radial cracks along the projectile path of a 9 mm Luger through a gelatin-filled head model. This is an oblique sagittal view of the head phantom in a transparent representation (**a**). The added computed tomography slice (**b**) shows the projectile path together with the cracks as a cross-sectional image at the position visualized by the red frame.

This study demonstrated that the effects of different ammunition types in gelatin-based head phantoms can be radiologically evaluated using CT-based crack length measurements (Fig. 4).

There is a direct correlation between the total crack length (TCL) in gelatin and the amount of energy transferred by a projectile [1]. When a projectile penetrates gelatin, it creates a temporary cavity that subsequently collapses due to the material’s elasticity, mimicking the behavior of biological soft tissue. Studies have shown that the sum of cracks radiating from the wound channel in gelatin is proportional to the volume of this cavity and, therefore, to the energy transferred per unit length [1]. The extent of the temporary cavity in the head phantoms used in this study, which is proportional to energy transfer, can be inferred from TCL measurements on CT. The high resolution of the CT images and the thin, uniform virtual slices along the projectile path allowed for precise crack length measurements. This CT-based method enables accurate TCL measurement in a straightforward manner, facilitating the creation of TCL curves to ultimately assess injury potential. Neither the sometimes significant destruction of the gelatin nor the presence of metal artifacts from projectile fragments along the wound channel substantially impeded measurements.

The primary objective of this study was to assess the feasibility of applying the CT-based TCL method to gelatin-based head phantoms, and specifically, the ability to generate TCL curves from CT-derived measurements of head phantoms impacted by projectiles. Therefore, the primary focus was on validating the application of CT-based TCL measurements in gelatin-based head phantoms rather than conducting a thorough scientific evaluation of wound ballistics. The resulting TCL curves serve as a supplement to existing literature.

Ballistic investigations into the effectiveness of different ammunition types in controlled simulant experiments are crucial for various fields, including forensic medicine, law enforcement, and military applications [17–22]. In forensic medicine, such studies assist in reconstructing gunshot wounds, aiding crime scene investigations and legal proceedings. In trauma medicine, they contribute to the development of improved surgical and emergency treatment strategies. For military and police forces, these experiments help assess the stopping power and penetration capabilities of different ammunition types, balancing effectiveness with minimizing collateral damage. Additionally, they play a critical role in the development and testing of new firearms, ammunition, and protective equipment such as ballistic vests.

The findings of this study support the idea that CT-based TCL measurements have the potential to enhance the accuracy and reliability of ballistic wound analysis while also simplifying the process. A CT-scanned simulant can be stored digitally and reanalyzed at any time, improving forensic reproducibility. Future research should explore the potential of modern CT scanners with higher image resolution, standardize measurement procedures, and integrate automated analysis tools to ensure consistency in forensic applications. Furthermore, incorporating CT diagnostics into experimental setups will aid in evaluating the accuracy of various simulants in mimicking biological tissue and identifying compositions that best replicate specific tissue types. Addressing these research areas would refine wound ballistics analysis and improve forensic reconstructions.

It is important to acknowledge the study’s limitations. The relatively small sample size of sixteen experiments using three different phantom models and six ammunition types constrains the statistical significance of the findings. As a result, statistical analysis could not be applied to assess projectile behavior at specific penetration depths; instead, only the rank sums of TCL values across the total penetration depth were compared. Additionally, the study included only two shooting distances (contact shots and 15-meter shots), which may not fully represent the variability of ballistic effects in real-world scenarios. In terms of data collection, all CT measurements and analyses were conducted by a single forensic pathologist, introducing the potential for observer bias and limiting reproducibility. The study also relied on specific imaging and measurement software, which may not be universally available in forensic or radiological settings. While metal artifacts did not significantly affect results in this study, they remain potential sources of measurement inaccuracy. These limitations underscore the need for further research involving larger sample sizes, standardized methodologies, and automated measurement techniques to enhance the reliability and applicability of CT-based TCL analysis in forensic and ballistic investigations.

## Conclusion

In conclusion, this study demonstrates the feasibility of using CT-based TCL measurements to assess the ballistic effects of different ammunition types in gelatin-based head phantoms. High-resolution CT imaging enabled precise quantification of crack lengths along the projectile path, offering insights into the transferred energy and injury potential of various projectiles. This method provides advantages over traditional slicing techniques, including non-destructive analysis and the ability to digitally store and reanalyze data. Future research should focus on standardizing measurement procedures, expanding sample sizes, and integrating automated analysis tools to enhance reliability and applicability in forensic and ballistic investigations. Ultimately, CT-based TCL analysis has the potential to advance the understanding of wound ballistics and contribute to forensic reconstructions, trauma research, and law enforcement applications.

## Key points

1. The CT data enables a ballistic phantom to be virtually cut into slices of the same thickness in cross-section along the bullet channel and thus contributes to the accuracy of the measurements.
2. The total crack length (TCL) in gelatin indirectly indicates the transferred energy of a penetrating bullet and can be measured on CT images.
3. Sixteen experiments with three types of gelatin phantoms and six types of ammunition showed statistically significant TCL differences.
4. Digital TCL analysis is feasible by CT scanning and thus this method supports forensic and ballistic investigations.

## Electronic supplementary material

Below is the link to the electronic supplementary material.

Supplementary Material 1

Supplementary Material 2

Supplementary Material 3

## Abbreviations

CT: Computed Tomography
FMJ: Full Metal Jacketed
GP: Gewehrpatrone
LR: Long Rifle
NATO: North Atlantic Treaty Organization
SJHP: Semi-Jacketed Hollow Point
TCL: Total Crack Length
