# Supplementary material for: Quantitative wound ballistic analysis of gelatin head phantoms by computed tomography using the total crack length method
Source: Forensic Sci Med Pathol. 2025 Mar 20;21(3):1248–55. doi: 10.1007/s12024-025-00995-9 (PMC12491355; doi:10.1007/s12024-025-00995-9)
Supplement: Supplementary file 1 — Supplementary Material 1 [file 12024_2025_995_MOESM1_ESM.pdf]

SUPPLEMENTARY MATERIAL

| **Internal study no.** | **Shot no.** | **Distance [m]** | **Weapon** | **Caliber [mm]** | **Projectile** | **Comment** | **Mass [g]** | **Velocity [$v_0$; m/s]** | **Muzzle Energy [J]** | **Phantom type** |
|---|---|---|---|---|---|---|---|---|---|---|
| 1 | 37 | 0 | Revolver | 10.9 | 44 Remington Magnum | Semi-jacketed hollow-point (SJHP) | 15.6 | - | - | 3 |
| 2 | 45 | 0 | Revolver | 10.9 | 44 Remington Magnum | Semi-jacketed hollow-point (SJHP) | 15.6 | - | - | 1 |
| 3 | - | 0 | Assault rifle | 5.56 | 5.56 mm NATO | Full metal jacketed spire-point (FMJSP) | 4.00 | - | - | 3 |
| 4 | - | 0 | Pistol | 9.0 | 9 mm Luger | Full metal jacketed round head (FMJ) | 8.00 | - | - | 1 |
| 5 | 36 | 0 | Pistol | 9.0 | 9 mm Luger | Full metal jacketed round head (FMJ) | 8.00 | - | - | 3 |
| 6 | - | 0 | Pistol | 9.0 | 9 mm Luger | Action 4 | 6.10 | - | - | 1 |
| 7 | 46 | 0 | Assault rifle | 7.5 | 7.5 x 55 GP11 (Swiss) | Full metal jacketed spire-point (FMJSP) | 11.30 | - | - | 1 |
| 8 | 43 | 0 | Rifle | 5.6 | 22 Long Rifle | Lead round nose (LRN) | 2.59 | - | - | 1 |
| 9 | 39 | 0 | Rifle | 5.6 | 22 Long Rifle | Lead round nose (LRN) | 2.59 | - | - | 2 |
| 10 | 35 | 15 | Test barrel | 5.56 | 5.56 mm NATO | Full metal jacketed spire-point (FMJSP) | 4.00 | 978 | 1913 | 1 |
| 11 | 10 | 15 | Test barrel | 9.0 | 9 mm Luger | Full metal jacketed round head (FMJ) | 8.00 | 356 | 507 | 1 |
| 12 | 9 | 15 | Test barrel | 9.0 | 9 mm Luger | Full metal jacketed round head (FMJ) | 8.00 | 365 | 533 | 2 |
| 13 | 11 | 15 | Test barrel | 9.0 | 9-mm Luger | Action 4 | 6.10 | 430 | 564 | 1 |
| 14 | 31 | 15 | Test barrel | 7.5 | 7.5 x 55 GP11 (Swiss) | Full metal jacketed spire-point (FMJSP) | 11.30 | 804 | 3652 | 1 |
| 15 | 30 | 15 | Test barrel | 7.5 | 7.5 x 55 GP11 (Swiss) | Full metal jacketed spire-point (FMJSP) | 11.30 | 799 | 3607 | 2 |
| 16 | 25 | 15 | Test barrel | 5.6 | 22 Long Rifle | Lead round nose (LRN) | 2.59 | 363 | 169 | 2 |

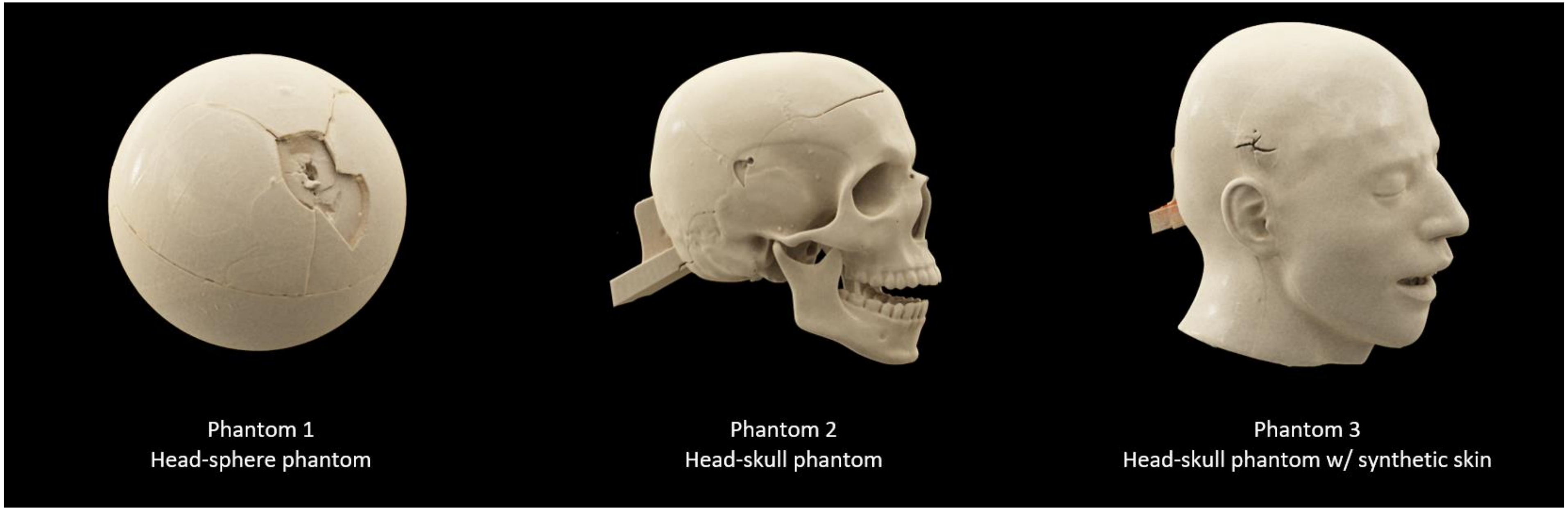
Phantom 1
Head-sphere phantom
Phantom 2
Head-skull phantom
Phantom 3
Head-skull phantom w/ synthetic skin
