# Supplementary material for: Quantitative wound ballistic analysis of gelatin head phantoms by computed tomography using the total crack length method
Source: Forensic Sci Med Pathol. 2025 Mar 20;21(3):1248–55. doi: 10.1007/s12024-025-00995-9 (PMC12491355; doi:10.1007/s12024-025-00995-9)
Supplement: Supplementary file 2 — Supplementary Material 2 [file 12024_2025_995_MOESM2_ESM.pdf]

<u>Description of the Measurement Procedure Using *SyngoVia* Software</u>

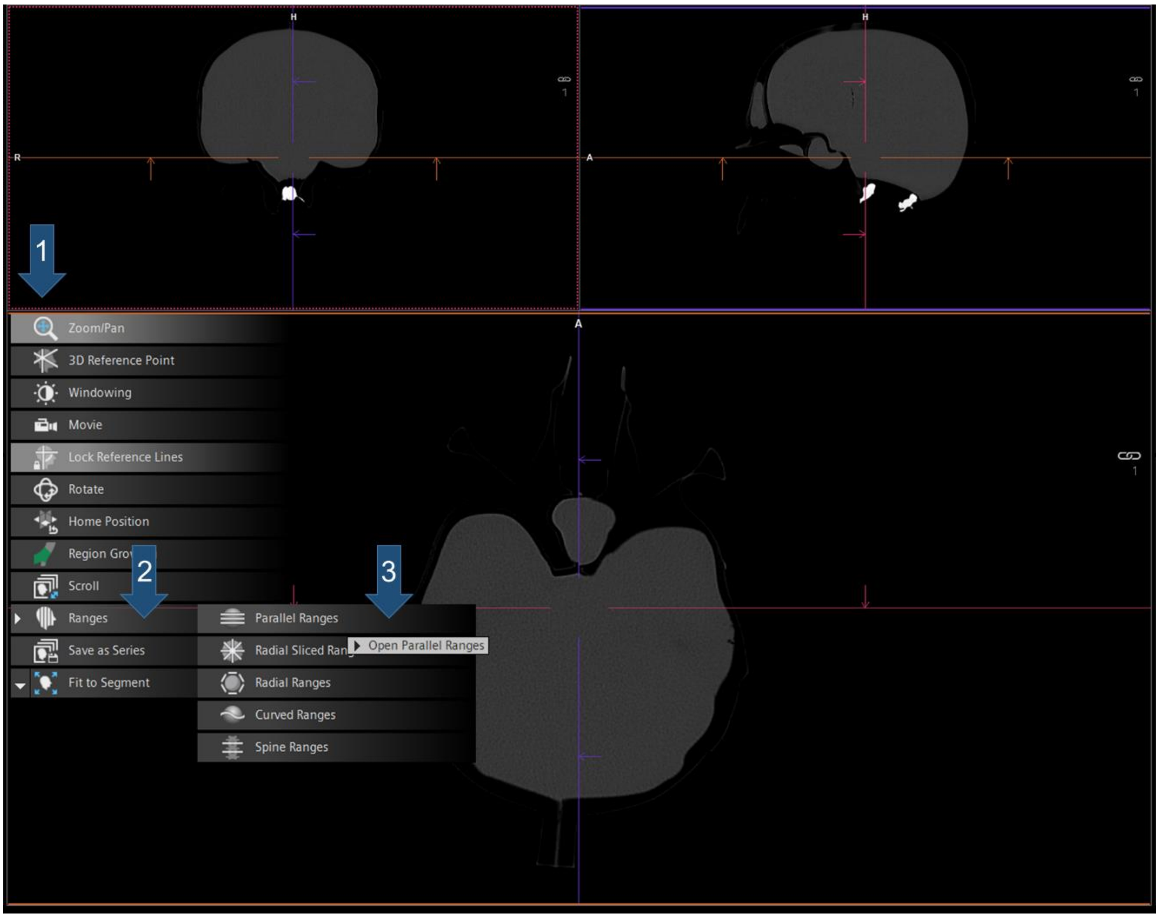

- The CT dataset is opened in SyngoVia as a multiplanar reconstruction and appears in three views: coronal (top left), sagittal (top right), and transverse (large image at the bottom).
- From the functions accessible via the upper left corner of each view (arrow 1), "Ranges" (arrow 2) and "Parallel Ranges" (arrow 3) is selected.

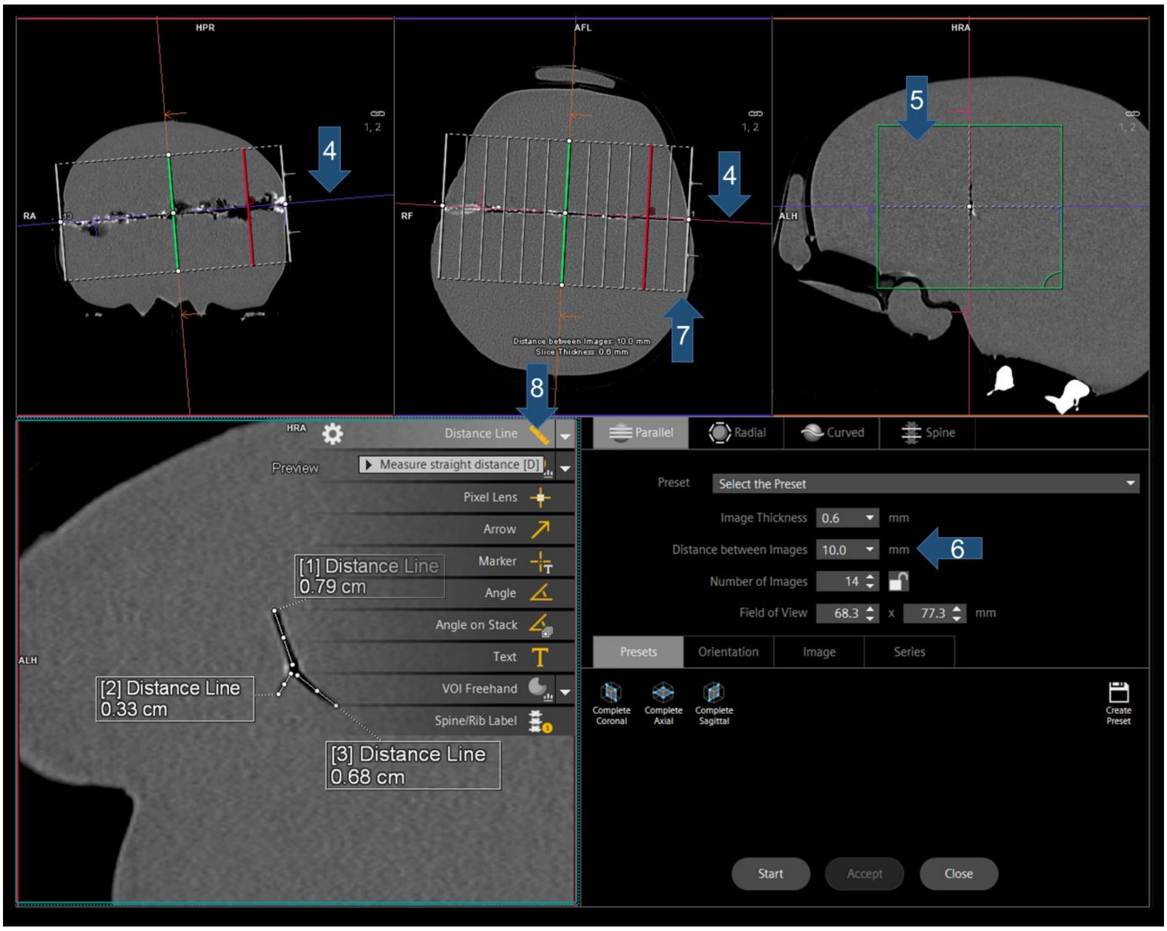

- In the Parallel Ranges workflow, the coronal, sagittal, and transverse views have to be aligned manually along the bullet path (arrow 4) to generate a cross-sectional view of the bullet trajectory (arrow 5). The cross-sectional view can be enlarged (the green frame in the top right image corresponds to the zoomed-in view shown in the bottom left image).
- Before starting the measurements, the image spacing is set to 10 mm (arrow 6). The positioning of the images is adjusted so that the first image is placed directly at the entry point of the projectile into the gelatin (arrow 7).
- In the magnified view, the "Distance Line" measurement tool is selected from the upper right corner (arrow 6). This tool is used to measure the length of the individual cracks in each image. Due to the distances between the images (1 cm), the increasing image number corresponds to the penetration depth in integer centimeter steps
