# Supplementary material for: Quantitative wound ballistic analysis of gelatin head phantoms by computed tomography using the total crack length method
Source: Forensic Sci Med Pathol. 2025 Mar 20;21(3):1248–55. doi: 10.1007/s12024-025-00995-9 (PMC12491355; doi:10.1007/s12024-025-00995-9)
Supplement: Supplementary file 3 — Supplementary Material 3 [file 12024_2025_995_MOESM3_ESM.pdf]

Dunn’s test results with the Bonferroni correction

.y.	group1	group2	n1	n2	statistic	p	p.adj	p.adj.signif
TCL	.22 LR LNR (1) C	.44 Rem Mag SJHP (3) C	17	14	5.318562504	1.0459E-07	1.25508E-05	****
TCL	.22 LR LNR (1) C	5.56 mm NATO (1) D	17	17	4.989597128	6.05053E-07	7.26064E-05	****
TCL	.22 LR LNR (1) C	.44 Rem Mag SJHP (1) C	17	15	4.941732637	7.74314E-07	9.29176E-05	****
TCL	.22 LR LNR (2) C	.44 Rem Mag SJHP (3) C	13	14	5.58192798	2.37867E-08	2.8544E-06	****
TCL	.22 LR LNR (2) C	5.56 mm NATO (1) D	13	17	5.270585797	1.35989E-07	1.63187E-05	****
TCL	.22 LR LNR (2) C	.44 Rem Mag SJHP (1) C	13	15	5.227992273	1.71361E-07	2.05633E-05	****
TCL	.22 LR LNR (2) C	5.56 mm NATO (3) C	13	15	4.975831232	6.49682E-07	7.79619E-05	****
TCL	.22 LR LNR (2) D	.44 Rem Mag SJHP (3) C	8	14	5.437287849	5.40977E-08	6.49172E-06	****
TCL	.22 LR LNR (2) D	5.56 mm NATO (1) D	8	17	5.135306542	2.81685E-07	3.38021E-05	****
TCL	.22 LR LNR (2) D	.44 Rem Mag SJHP (1) C	8	15	5.118611964	3.07792E-07	3.69351E-05	****
TCL	.44 Rem Mag SJHP (1) C	9 mm Luger FMJ (2) D	15	14	-5.036917833	4.73088E-07	5.67705E-05	****
TCL	.44 Rem Mag SJHP (3) C	9 mm Luger FMJ (2) D	14	14	-5.399137455	6.69621E-08	8.03545E-06	****
TCL	5.56 mm NATO (1) D	9 mm Luger FMJ (2) D	17	14	-5.077814442	3.81801E-07	4.58162E-05	****
TCL	.22 LR LNR (1) C	5.56 mm NATO (3) C	17	15	4.671999191	2.98282E-06	0.000357939	***
TCL	.22 LR LNR (2) D	5.56 mm NATO (3) C	8	15	4.900355857	9.56632E-07	0.000114796	***
TCL	.44 Rem Mag SJHP (3) C	7.5 mm GP11 (1) C	14	18	-4.803883244	1.55618E-06	0.000186741	***
TCL	.44 Rem Mag SJHP (3) C	9 mm Luger FMJ (1) D	14	15	-4.55134623	5.33037E-06	0.000639645	***
TCL	5.56 mm NATO (3) C	9 mm Luger FMJ (2) D	15	14	-4.779789256	1.75479E-06	0.000210575	***
TCL	.22 LR LNR (2) D	9 mm Luger Action 4 (1) C	8	17	3.951385882	7.76999E-05	0.009323991	**
TCL	.44 Rem Mag SJHP (1) C	7.5 mm GP11 (1) C	15	18	-4.413438375	1.01742E-05	0.0012209	**
TCL	.44 Rem Mag SJHP (1) C	9 mm Luger FMJ (1) D	15	15	-4.169342815	3.05479E-05	0.003665751	**
TCL	.44 Rem Mag SJHP (3) C	9 mm Luger FMJ (3) C	14	13	-4.152166921	3.29342E-05	0.003952103	**
TCL	5.56 mm NATO (1) D	7.5 mm GP11 (1) C	17	18	-4.446425176	8.73111E-06	0.001047733	**
TCL	5.56 mm NATO (1) D	9 mm Luger FMJ (1) D	17	15	-4.187084213	2.82561E-05	0.003390732	**
TCL	5.56 mm NATO (3) C	7.5 mm GP11 (1) C	15	18	-4.140122655	3.4712E-05	0.004165442	**

TCL	.22 LR LNR (2) C	9 mm Luger Action 4 (1) C	13	17	3.892873107	9.9064E-05	0.011887677	*
TCL	.44 Rem Mag SJHP (1) C	9 mm Luger FMJ (3) C	15	13	-3.774716804	0.00016019	0.019222745	*
TCL	.44 Rem Mag SJHP (3) C	9 mm Luger FMJ (1) C	14	17	-3.881987249	0.000103606	0.012432757	*
TCL	.44 Rem Mag SJHP (3) C	7.5 mm GP11 (2) D	14	14	-3.627349136	0.000286346	0.034361518	*
TCL	.44 Rem Mag SJHP (3) C	9 mm Luger Action 4 (1) D	14	16	-3.585707582	0.000336165	0.040339857	*
TCL	5.56 mm NATO (1) D	9 mm Luger FMJ (3) C	17	13	-3.775916333	0.000159421	0.01913046	*
TCL	5.56 mm NATO (3) C	9 mm Luger FMJ (1) D	15	15	-3.907662936	9.31932E-05	0.011183183	*
TCL	9 mm Luger Action 4 (1) C	9 mm Luger FMJ (2) D	17	14	-3.671343445	0.000241279	0.028953463	*
TCL	.22 LR LNR (1) C	9 mm Luger Action 4 (1) C	17	17	3.509696304	0.000448619	0.053834252	ns
TCL	.22 LR LNR (1) C	.22 LR LNR (2) C	17	13	-0.625523662	0.531627423	1	ns
TCL	.22 LR LNR (1) C	.22 LR LNR (2) D	17	8	-1.143628839	0.252777596	1	ns
TCL	.22 LR LNR (1) C	7.5 mm GP11 (1) C	17	18	0.613949915	0.539248424	1	ns
TCL	.22 LR LNR (1) C	7.5 mm GP11 (1) D	17	11	2.191697105	0.028401385	1	ns
TCL	.22 LR LNR (1) C	7.5 mm GP11 (2) D	17	14	1.519749024	0.128574065	1	ns
TCL	.22 LR LNR (1) C	9 mm Luger Action 4 (1) D	17	16	1.743417226	0.081260762	1	ns
TCL	.22 LR LNR (1) C	9 mm Luger FMJ (1) C	17	17	1.511576784	0.130641562	1	ns
TCL	.22 LR LNR (1) C	9 mm Luger FMJ (1) D	17	15	0.644072432	0.519528466	1	ns
TCL	.22 LR LNR (1) C	9 mm Luger FMJ (2) D	17	14	-0.33579151	0.737028093	1	ns
TCL	.22 LR LNR (1) C	9 mm Luger FMJ (3) C	17	13	0.869145802	0.384767387	1	ns
TCL	.22 LR LNR (2) C	.22 LR LNR (2) D	13	8	-0.578291238	0.563067509	1	ns
TCL	.22 LR LNR (2) C	7.5 mm GP11 (1) C	13	18	1.203662536	0.228720038	1	ns
TCL	.22 LR LNR (2) C	7.5 mm GP11 (1) D	13	11	2.632709111	0.008470686	1	ns
TCL	.22 LR LNR (2) C	7.5 mm GP11 (2) D	13	14	2.022385741	0.043136517	1	ns
TCL	.22 LR LNR (2) C	9 mm Luger Action 4 (1) D	13	16	2.243543229	0.024861805	1	ns
TCL	.22 LR LNR (2) C	9 mm Luger FMJ (1) C	13	17	2.032725066	0.042080307	1	ns
TCL	.22 LR LNR (2) C	9 mm Luger FMJ (1) D	13	15	1.210313006	0.226158809	1	ns
TCL	.22 LR LNR (2) C	9 mm Luger FMJ (2) D	13	14	0.283717881	0.776626591	1	ns
TCL	.22 LR LNR (2) C	9 mm Luger FMJ (3) C	13	13	1.403997488	0.160319597	1	ns
TCL	.22 LR LNR (2) D	7.5 mm GP11 (1) D	8	11	2.880407255	0.003971618	0.47659414	ns
TCL	.22 LR LNR (2) D	7.5 mm GP11 (1) C	8	18	1.642585297	0.100468765	1	ns
TCL	.22 LR LNR (2) D	7.5 mm GP11 (2) D	8	14	2.343874111	0.019084612	1	ns

TCL	.22 LR LNR (2) D	9 mm Luger Action 4 (1) D	8	16	2.53476552	0.011252262	1	ns
TCL	.22 LR LNR (2) D	9 mm Luger FMJ (1) C	8	17	2.352890266	0.01862813	1	ns
TCL	.22 LR LNR (2) D	9 mm Luger FMJ (1) D	8	15	1.641139668	0.100768426	1	ns
TCL	.22 LR LNR (2) D	9 mm Luger FMJ (2) D	8	14	0.832887956	0.404907923	1	ns
TCL	.22 LR LNR (2) D	9 mm Luger FMJ (3) C	8	13	1.803800717	0.071262553	1	ns
TCL	.44 Rem Mag SJHP (1) C	9 mm Luger FMJ (1) C	15	17	-3.478154709	0.000504878	0.060585412	ns
TCL	.44 Rem Mag SJHP (1) C	7.5 mm GP11 (2) D	15	14	-3.23484034	0.001217108	0.146052923	ns
TCL	.44 Rem Mag SJHP (1) C	9 mm Luger Action 4 (1) D	15	16	-3.181239401	0.001466464	0.17597567	ns
TCL	.44 Rem Mag SJHP (1) C	.44 Rem Mag SJHP (3) C	15	14	0.454519256	0.649455135	1	ns
TCL	.44 Rem Mag SJHP (1) C	5.56 mm NATO (1) D	15	17	-0.110575991	0.911952587	1	ns
TCL	.44 Rem Mag SJHP (1) C	5.56 mm NATO (3) C	15	15	-0.261679879	0.793568254	1	ns
TCL	.44 Rem Mag SJHP (1) C	7.5 mm GP11 (1) D	15	11	-2.273552208	0.022992924	1	ns
TCL	.44 Rem Mag SJHP (1) C	9 mm Luger Action 4 (1) C	15	17	-1.543483803	0.122713435	1	ns
TCL	.44 Rem Mag SJHP (3) C	7.5 mm GP11 (1) D	14	11	-2.659168937	0.007833367	0.940004082	ns
TCL	.44 Rem Mag SJHP (3) C	5.56 mm NATO (1) D	14	17	-0.576539573	0.56425053	1	ns
TCL	.44 Rem Mag SJHP (3) C	5.56 mm NATO (3) C	14	15	-0.711647834	0.476682879	1	ns
TCL	.44 Rem Mag SJHP (3) C	9 mm Luger Action 4 (1) C	14	17	-1.983010569	0.047366249	1	ns
TCL	5.56 mm NATO (1) D	9 mm Luger FMJ (1) C	17	17	-3.478020345	0.000505132	0.06061579	ns
TCL	5.56 mm NATO (1) D	7.5 mm GP11 (2) D	17	14	-3.222273908	0.001271775	0.152612991	ns
TCL	5.56 mm NATO (1) D	9 mm Luger Action 4 (1) D	17	16	-3.169998374	0.001524398	0.182927749	ns
TCL	5.56 mm NATO (1) D	5.56 mm NATO (3) C	17	15	-0.159157454	0.873544823	1	ns
TCL	5.56 mm NATO (1) D	7.5 mm GP11 (1) D	17	11	-2.231108037	0.025673973	1	ns
TCL	5.56 mm NATO (1) D	9 mm Luger Action 4 (1) C	17	17	-1.479900824	0.138899716	1	ns
TCL	5.56 mm NATO (3) C	9 mm Luger FMJ (3) C	15	13	-3.522555763	0.000427407	0.051288854	ns
TCL	5.56 mm NATO (3) C	9 mm Luger FMJ (1) C	15	17	-3.208421264	0.001334658	0.160159018	ns
TCL	5.56 mm NATO (3) C	7.5 mm GP11 (2) D	15	14	-2.977711762	0.00290409	0.348490742	ns
TCL	5.56 mm NATO (3) C	9 mm Luger Action 4 (1) D	15	16	-2.915372378	0.003552644	0.426317248	ns
TCL	5.56 mm NATO (3) C	7.5 mm GP11 (1) D	15	11	-2.032841729	0.042068516	1	ns
TCL	5.56 mm NATO (3) C	9 mm Luger Action 4 (1) C	15	17	-1.273750357	0.202751904	1	ns
TCL	7.5 mm GP11 (1) C	9 mm Luger Action 4 (1) C	18	17	2.945531802	0.003224	0.386880004	ns
TCL	7.5 mm GP11 (1) C	7.5 mm GP11 (1) D	18	11	1.673463096	0.094236166	1	ns

TCL	7.5 mm GP11 (1) C	7.5 mm GP11 (2) D	18	14	0.956498486	0.338820449	1	ns
TCL	7.5 mm GP11 (1) C	9 mm Luger Action 4 (1) D	18	16	1.163068316	0.244801786	1	ns
TCL	7.5 mm GP11 (1) C	9 mm Luger FMJ (1) C	18	17	0.919068745	0.358059616	1	ns
TCL	7.5 mm GP11 (1) C	9 mm Luger FMJ (1) D	18	15	0.05870183	0.953189602	1	ns
TCL	7.5 mm GP11 (1) C	9 mm Luger FMJ (2) D	18	14	-0.922766817	0.356128737	1	ns
TCL	7.5 mm GP11 (1) C	9 mm Luger FMJ (3) C	18	13	0.309329822	0.757070648	1	ns
TCL	7.5 mm GP11 (1) D	7.5 mm GP11 (2) D	11	14	-0.743586174	0.457126871	1	ns
TCL	7.5 mm GP11 (1) D	9 mm Luger Action 4 (1) C	11	17	0.919316162	0.357930227	1	ns
TCL	7.5 mm GP11 (1) D	9 mm Luger Action 4 (1) D	11	16	-0.614859463	0.538647535	1	ns
TCL	7.5 mm GP11 (1) D	9 mm Luger FMJ (1) C	11	17	-0.851827492	0.394309845	1	ns
TCL	7.5 mm GP11 (1) D	9 mm Luger FMJ (1) D	11	15	-1.56168537	0.118362127	1	ns
TCL	7.5 mm GP11 (1) D	9 mm Luger FMJ (2) D	11	14	-2.405670945	0.016142798	1	ns
TCL	7.5 mm GP11 (1) D	9 mm Luger FMJ (3) C	11	13	-1.288483856	0.197577585	1	ns
TCL	7.5 mm GP11 (2) D	9 mm Luger Action 4 (1) C	14	17	1.815802911	0.069400611	1	ns
TCL	7.5 mm GP11 (2) D	9 mm Luger Action 4 (1) D	14	16	0.160602496	0.872406489	1	ns
TCL	7.5 mm GP11 (2) D	9 mm Luger FMJ (1) C	14	17	-0.083173768	0.933713371	1	ns
TCL	7.5 mm GP11 (2) D	9 mm Luger FMJ (1) D	14	15	-0.861986634	0.388694873	1	ns
TCL	7.5 mm GP11 (2) D	9 mm Luger FMJ (2) D	14	14	-1.771788319	0.076429702	1	ns
TCL	7.5 mm GP11 (2) D	9 mm Luger FMJ (3) C	14	13	-0.592624682	0.553432357	1	ns
TCL	9 mm Luger Action 4 (1) C	9 mm Luger FMJ (1) D	17	15	-2.754176402	0.005884004	0.706080537	ns
TCL	9 mm Luger Action 4 (1) C	9 mm Luger Action 4 (1) D	17	16	-1.712692782	0.086769074	1	ns
TCL	9 mm Luger Action 4 (1) C	9 mm Luger FMJ (1) C	17	17	-1.99811952	0.045703704	1	ns
TCL	9 mm Luger Action 4 (1) C	9 mm Luger FMJ (3) C	17	13	-2.398203643	0.016475703	1	ns
TCL	9 mm Luger Action 4 (1) D	9 mm Luger FMJ (1) C	16	17	-0.254919305	0.798785425	1	ns
TCL	9 mm Luger Action 4 (1) D	9 mm Luger FMJ (1) D	16	15	-1.054817135	0.291508966	1	ns
TCL	9 mm Luger Action 4 (1) D	9 mm Luger FMJ (2) D	16	14	-1.990497604	0.046536148	1	ns
TCL	9 mm Luger Action 4 (1) D	9 mm Luger FMJ (3) C	16	13	-0.768712013	0.442064292	1	ns
TCL	9 mm Luger FMJ (1) C	9 mm Luger FMJ (1) D	17	15	-0.819505495	0.412498068	1	ns
TCL	9 mm Luger FMJ (1) C	9 mm Luger FMJ (2) D	17	14	-1.772366766	0.076333695	1	ns
TCL	9 mm Luger FMJ (1) C	9 mm Luger FMJ (3) C	17	13	-0.538055601	0.590538663	1	ns
TCL	9 mm Luger FMJ (1) D	9 mm Luger FMJ (2) D	15	14	-0.940090859	0.347170957	1	ns

TCL	9 mm Luger FMJ (1) D	9 mm Luger FMJ (3) C	15	13	0.242962462	0.808034477	1		ns
TCL	9 mm Luger FMJ (2) D	9 mm Luger FMJ (3) C	14	13	1.146043178	0.251777287	1		ns

.y. = test variable, n = number of values, p.adj = p-value adjusted with the Bonferroni correction, TCL = Total Crack length, LR = long rifle, LNR = lead round nose, SJHP = semi-jacketed hollow point, NATO = North Atlantic Treaty Organization, GP = Gewehrpatrone, FMJ = full metal jacket, ns = not significant ($p > 0.05$), * = $p \leq 0.05$, ** = $p \leq 0.01$, *** = $p \leq 0.001$, **** = $p \leq 0.0001$. Note: The number in brackets next to the projectile designation indicates the phantom: 1 = spherical head phantom, 2 = skull phantom, 3 = skull phantom with skin simulant. The letter C or D next to the phantom specification stands for contact shot (= C) and long-range or distance shot (= D).
